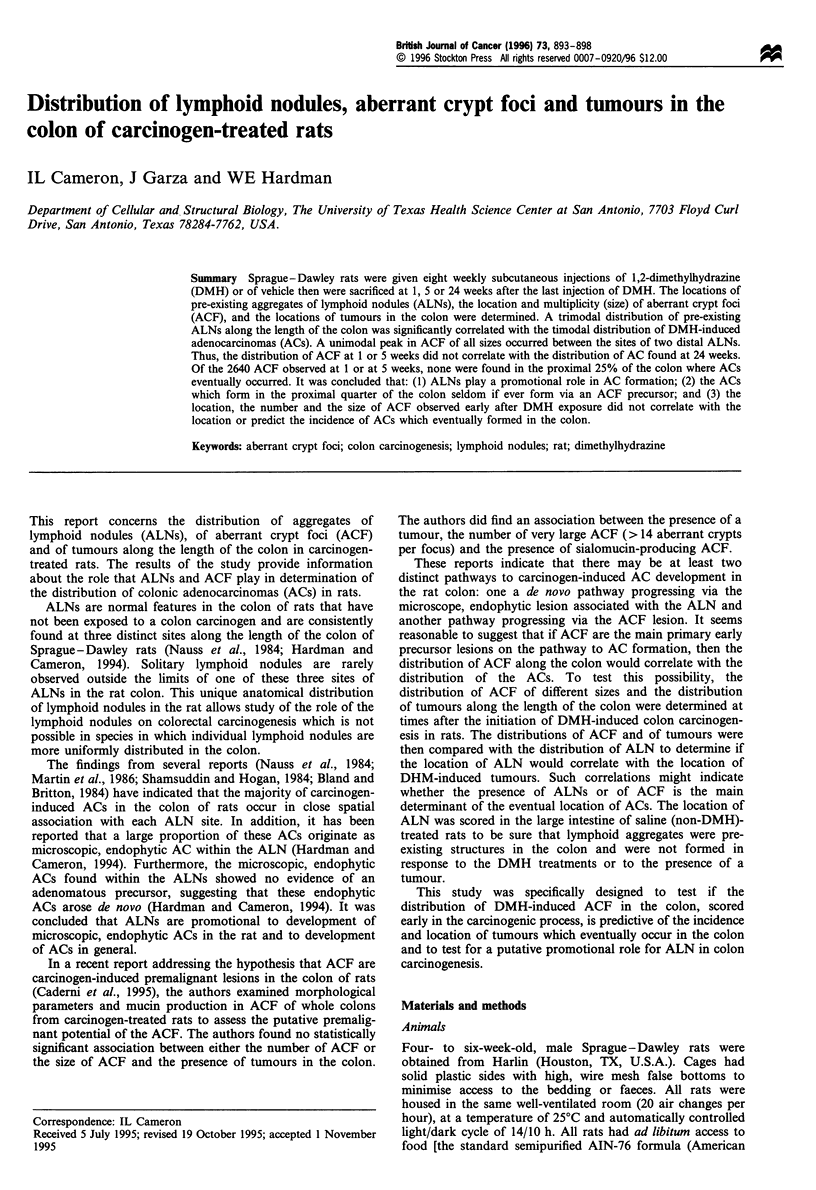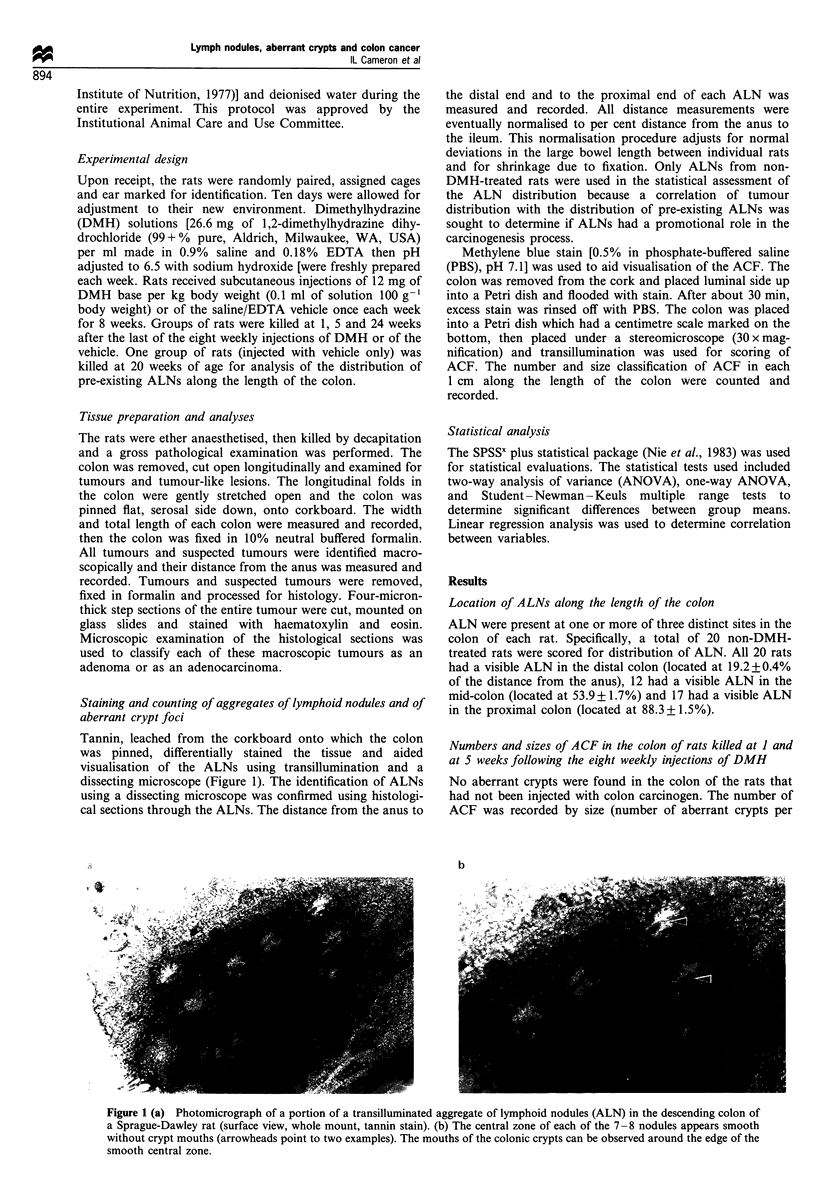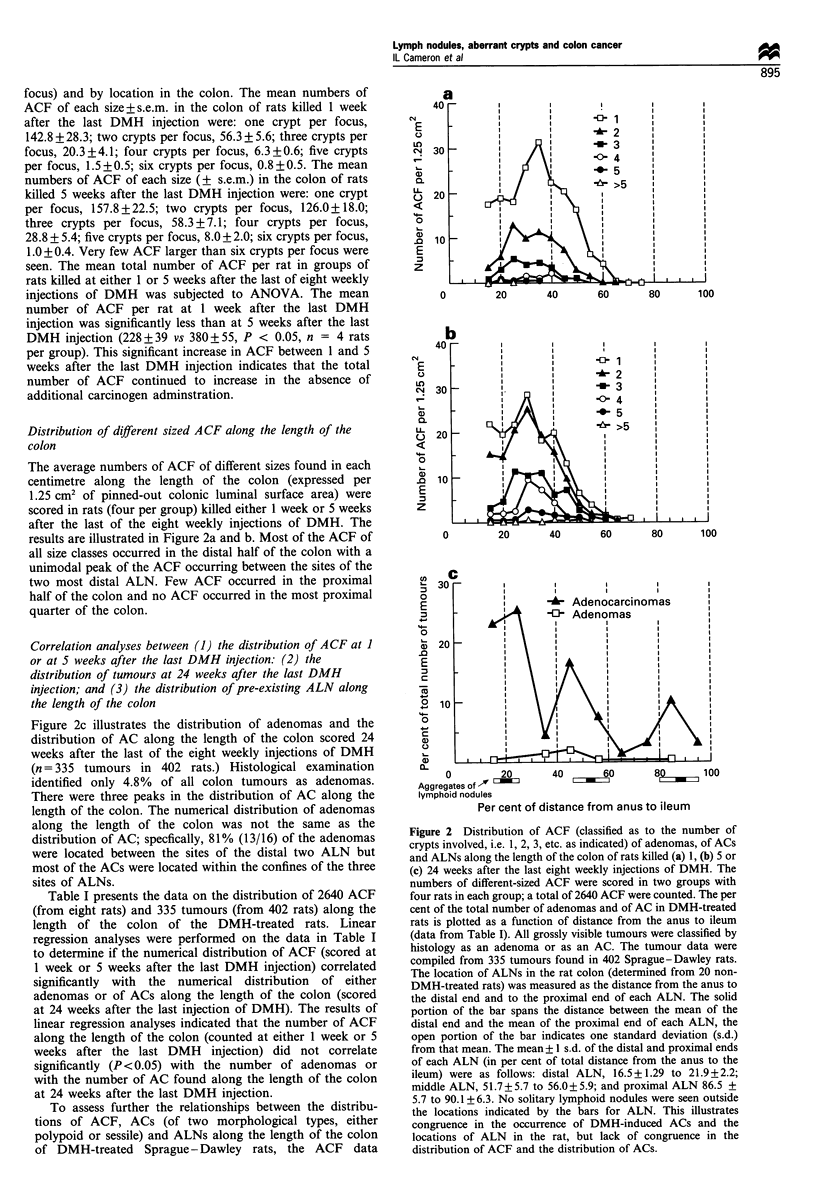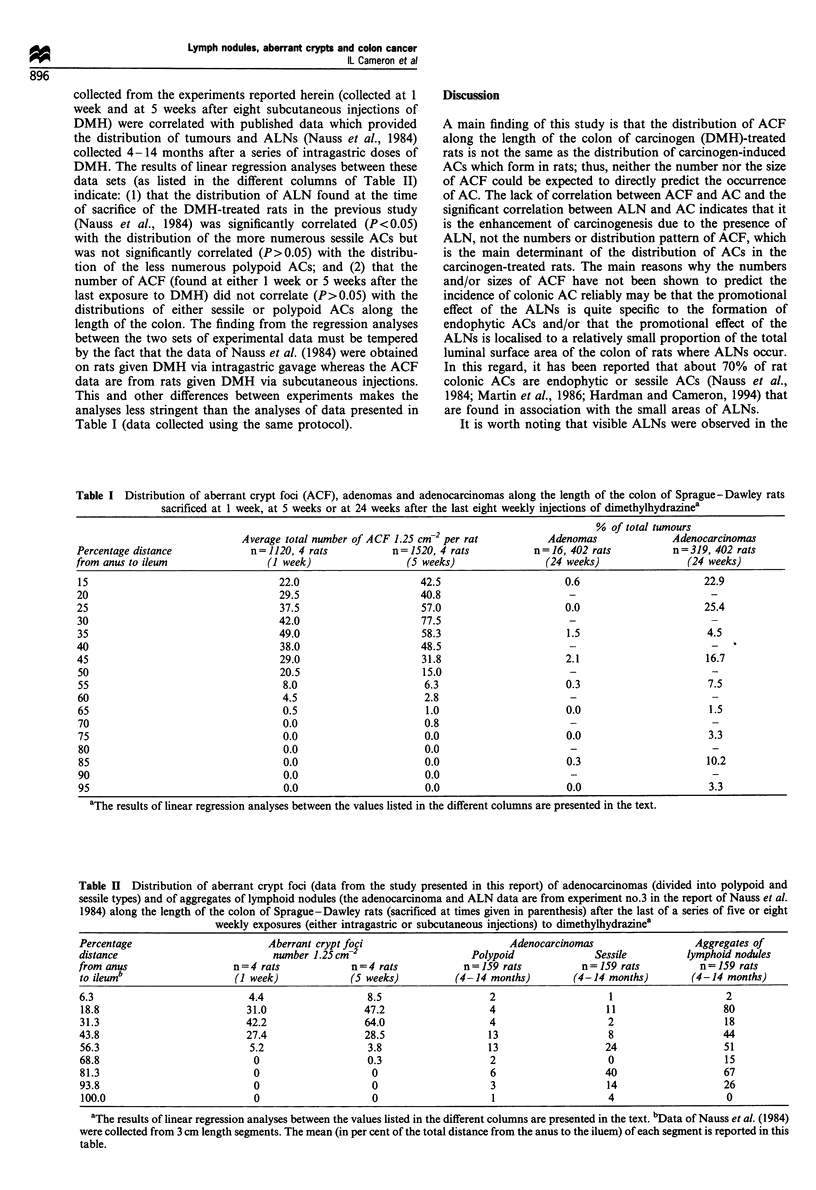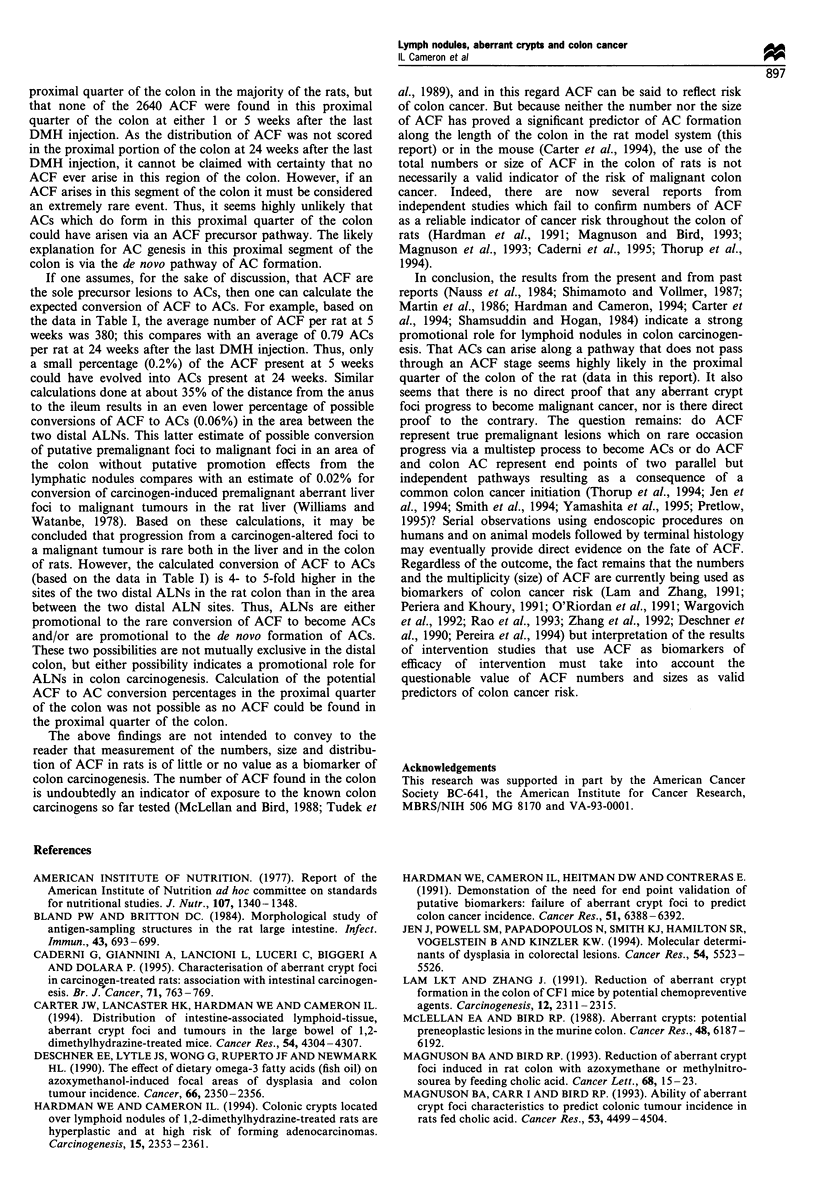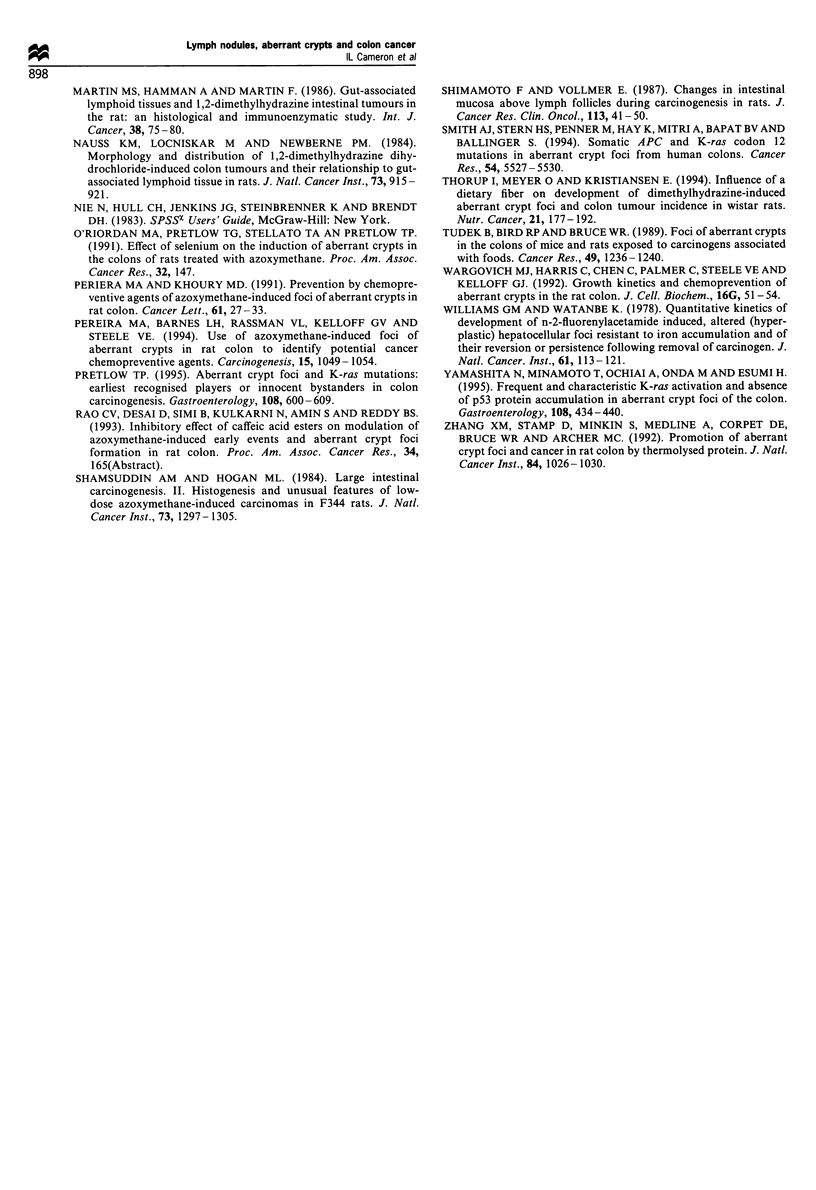# Distribution of lymphoid nodules, aberrant crypt foci and tumours in the colon of carcinogen-treated rats.

**DOI:** 10.1038/bjc.1996.159

**Published:** 1996-04

**Authors:** I. L. Cameron, J. Garza, W. E. Hardman

**Affiliations:** Department of Cellular and Structural Biology, The University of Texas Health Science Center at San Antonio, 78284-7762, USA.

## Abstract

**Images:**


British Journal of Cancer (1996) 73, 893–898
© 1996 Stockton Press All rights reserved 0007–0920/96 $12.00

### Distribution of lymphoid nodules, aberrant crypt foci and tumours in the colon of carcinogen-treated rats

IL Cameron, J Garza and WE Hardman

*Department of Cellular and Structural Biology, The University of Texas Health Science Center at San Antonio, 7703 Floyd Curl Drive, San Antonio, Texas 78284-7762, USA.*

**Summary** Sprague–Dawley rats were given eight weekly subcutaneous injections of 1,2-dimethylhydrazine (DMH) or of vehicle then were sacrificed at 1, 5 or 24 weeks after the last injection of DMH. The locations of pre-existing aggregates of lymphoid nodules (ALNs), the location and multiplicity (size) of aberrant crypt foci (ACF), and the locations of tumours in the colon were determined. A trimodal distribution of pre-existing ALNs along the length of the colon was significantly correlated with the timodal distribution of DMH-induced adenocarcinomas (ACs). A unimodal peak in ACF of all sizes occurred between the sites of two distal ALNs. Thus, the distribution of ACF at 1 or 5 weeks did not correlate with the distribution of AC found at 24 weeks. Of the 2640 ACF observed at 1 or at 5 weeks, none were found in the proximal 25% of the colon where ACs eventually occurred. It was concluded that: (1) ALNs play a promotional role in AC formation; (2) the ACs which form in the proximal quarter of the colon seldom if ever form via an ACF precursor; and (3) the location, the number and the size of ACF observed early after DMH exposure did not correlate with the location or predict the incidence of ACs which eventually formed in the colon.

**Keywords:** aberrant crypt foci; colon carcinogenesis; lymphoid nodules; rat; dimethylhydrazine

This report concerns the distribution of aggregates of lymphoid nodules (ALNs), of aberrant crypt foci (ACF) and of tumours along the length of the colon in carcinogen-treated rats. The results of the study provide information about the role that ALNs and ACF play in determination of the distribution of colonic adenocarcinomas (ACs) in rats.

ALNs are normal features in the colon of rats that have not been exposed to a colon carcinogen and are consistently found at three distinct sites along the length of the colon of Sprague–Dawley rats (Nauss *et al.*, 1984; Hardman and Cameron, 1994). Solitary lymphoid nodules are rarely observed outside the limits of one of these three sites of ALNs in the rat colon. This unique anatomical distribution of lymphoid nodules in the rat allows study of the role of the lymphoid nodules on colorectal carcinogenesis which is not possible in species in which individual lymphoid nodules are more uniformly distributed in the colon.

The findings from several reports (Nauss *et al.*, 1984; Martin *et al.*, 1986; Shamsuddin and Hogan, 1984; Bland and Britton, 1984) have indicated that the majority of carcinogen-induced ACs in the colon of rats occur in close spatial association with each ALN site. In addition, it has been reported that a large proportion of these ACs originate as microscopic, endophytic AC within the ALN (Hardman and Cameron, 1994). Furthermore, the microscopic, endophytic ACs found within the ALNs showed no evidence of an adenomatous precursor, suggesting that these endophytic ACs arose *de novo* (Hardman and Cameron, 1994). It was concluded that ALNs are promotional to development of microscopic, endophytic ACs in the rat and to development of ACs in general.

In a recent report addressing the hypothesis that ACF are carcinogen-induced premalignant lesions in the colon of rats (Caderni *et al.*, 1995), the authors examined morphological parameters and mucin production in ACF of whole colons from carcinogen-treated rats to assess the putative premalignant potential of the ACF. The authors found no statistically significant association between either the number of ACF or the size of ACF and the presence of tumours in the colon. The authors did find an association between the presence of a tumour, the number of very large ACF (>14 aberrant crypts per focus) and the presence of sialomucin-producing ACF.

These reports indicate that there may be at least two distinct pathways to carcinogen-induced AC development in the rat colon: one a *de novo* pathway progressing via the microscope, endophytic lesion associated with the ALN and another pathway progressing via the ACF lesion. It seems reasonable to suggest that if ACF are the main primary early precursor lesions on the pathway to AC formation, then the distribution of ACF along the colon would correlate with the distribution of the ACs. To test this possibility, the distribution of ACF of different sizes and the distribution of tumours along the length of the colon were determined at times after the initiation of DMH-induced colon carcinogenesis in rats. The distributions of ACF and of tumours were then compared with the distribution of ALN to determine if the location of ALN would correlate with the location of DHM-induced tumours. Such correlations might indicate whether the presence of ALNs or of ACF is the main determinant of the eventual location of ACs. The location of ALN was scored in the large intestine of saline (non-DMH)-treated rats to be sure that lymphoid aggregates were pre-existing structures in the colon and were not formed in response to the DMH treatments or to the presence of a tumour.

This study was specifically designed to test if the distribution of DMH-induced ACF in the colon, scored early in the carcinogenic process, is predictive of the incidence and location of tumours which eventually occur in the colon and to test for a putative promotional role for ALN in colon carcinogenesis.

### Materials and methods

### *Animals*

Four- to six-week-old, male Sprague–Dawley rats were obtained from Harlin (Houston, TX, U.S.A.). Cages had solid plastic sides with high, wire mesh false bottoms to minimise access to the bedding or faeces. All rats were housed in the same well-ventilated room (20 air changes per hour), at a temperature of 25°C and automatically controlled light/dark cycle of 14/10 h. All rats had *ad libitum* access to food [the standard semipurified AIN-76 formula (American

Correspondence: IL Cameron
Received 5 July 1995; revised 19 October 1995; accepted 1 November 1995

Institute of Nutrition, 1977)] and deionised water during the entire experiment. This protocol was approved by the Institutional Animal Care and Use Committee.

*Experimental design*

Upon receipt, the rats were randomly paired, assigned cages and ear marked for identification. Ten days were allowed for adjustment to their new environment. Dimethylhydrazine (DMH) solutions [26.6 mg of 1,2-dimethylhydrazine dihydrochloride (99+% pure, Aldrich, Milwaukee, WA, USA) per ml made in 0.9% saline and 0.18% EDTA then pH adjusted to 6.5 with sodium hydroxide [were freshly prepared each week. Rats received subcutaneous injections of 12 mg of DMH base per kg body weight (0.1 ml of solution 100 $g^{-1}$ body weight) or of the saline/EDTA vehicle once each week for 8 weeks. Groups of rats were killed at 1, 5 and 24 weeks after the last of the eight weekly injections of DMH or of the vehicle. One group of rats (injected with vehicle only) was killed at 20 weeks of age for analysis of the distribution of pre-existing ALNs along the length of the colon.

*Tissue preparation and analyses*

The rats were ether anaesthetised, then killed by decapitation and a gross pathological examination was performed. The colon was removed, cut open longitudinally and examined for tumours and tumour-like lesions. The longitudinal folds in the colon were gently stretched open and the colon was pinned flat, serosal side down, onto corkboard. The width and total length of each colon were measured and recorded, then the colon was fixed in 10% neutral buffered formalin. All tumours and suspected tumours were identified macroscopically and their distance from the anus was measured and recorded. Tumours and suspected tumours were removed, fixed in formalin and processed for histology. Four-micron-thick step sections of the entire tumour were cut, mounted on glass slides and stained with haematoxylin and eosin. Microscopic examination of the histological sections was used to classify each of these macroscopic tumours as an adenoma or as an adenocarcinoma.

*Staining and counting of aggregates of lymphoid nodules and of aberrant crypt foci*

Tannin, leached from the corkboard onto which the colon was pinned, differentially stained the tissue and aided visualisation of the ALNs using transillumination and a dissecting microscope (Figure 1). The identification of ALNs using a dissecting microscope was confirmed using histological sections through the ALNs. The distance from the anus to the distal end and to the proximal end of each ALN was measured and recorded. All distance measurements were eventually normalised to per cent distance from the anus to the ileum. This normalisation procedure adjusts for normal deviations in the large bowel length between individual rats and for shrinkage due to fixation. Only ALNs from non-DMH-treated rats were used in the statistical assessment of the ALN distribution because a correlation of tumour distribution with the distribution of pre-existing ALNs was sought to determine if ALNs had a promotional role in the carcinogenesis process.

Methylene blue stain [0.5% in phosphate-buffered saline (PBS), pH 7.1] was used to aid visualisation of the ACF. The colon was removed from the cork and placed luminal side up into a Petri dish and flooded with stain. After about 30 min, excess stain was rinsed off with PBS. The colon was placed into a Petri dish which had a centimetre scale marked on the bottom, then placed under a stereomicroscope (30 × magnification) and transillumination was used for scoring of ACF. The number and size classification of ACF in each 1 cm along the length of the colon were counted and recorded.

*Statistical analysis*

The SPSS$^x$ plus statistical package (Nie *et al.*, 1983) was used for statistical evaluations. The statistical tests used included two-way analysis of variance (ANOVA), one-way ANOVA, and Student–Newman–Keuls multiple range tests to determine significant differences between group means. Linear regression analysis was used to determine correlation between variables.

### Results

*Location of ALNs along the length of the colon*

ALN were present at one or more of three distinct sites in the colon of each rat. Specifically, a total of 20 non-DMH-treated rats were scored for distribution of ALN. All 20 rats had a visible ALN in the distal colon (located at $19.2 \pm 0.4\%$ of the distance from the anus), 12 had a visible ALN in the mid-colon (located at $53.9 \pm 1.7\%$) and 17 had a visible ALN in the proximal colon (located at $88.3 \pm 1.5\%$).

*Numbers and sizes of ACF in the colon of rats killed at 1 and at 5 weeks following the eight weekly injections of DMH*

No aberrant crypts were found in the colon of the rats that had not been injected with colon carcinogen. The number of ACF was recorded by size (number of aberrant crypts per

**Figure 1 (a)** Photomicrograph of a portion of a transilluminated aggregate of lymphoid nodules (ALN) in the descending colon of a Sprague-Dawley rat (surface view, whole mount, tannin stain). (b) The central zone of each of the 7–8 nodules appears smooth without crypt mouths (arrowheads point to two examples). The mouths of the colonic crypts can be observed around the edge of the smooth central zone.

focus) and by location in the colon. The mean numbers of ACF of each size ± s.e.m. in the colon of rats killed 1 week after the last DMH injection were: one crypt per focus, 142.8 ± 28.3; two crypts per focus, 56.3 ± 5.6; three crypts per focus, 20.3 ± 4.1; four crypts per focus, 6.3 ± 0.6; five crypts per focus, 1.5 ± 0.5; six crypts per focus, 0.8 ± 0.5. The mean numbers of ACF of each size (± s.e.m.) in the colon of rats killed 5 weeks after the last DMH injection were: one crypt per focus, 157.8 ± 22.5; two crypts per focus, 126.0 ± 18.0; three crypts per focus, 58.3 ± 7.1; four crypts per focus, 28.8 ± 5.4; five crypts per focus, 8.0 ± 2.0; six crypts per focus, 1.0 ± 0.4. Very few ACF larger than six crypts per focus were seen. The mean total number of ACF per rat in groups of rats killed at either 1 or 5 weeks after the last of eight weekly injections of DMH was subjected to ANOVA. The mean number of ACF per rat at 1 week after the last DMH injection was significantly less than at 5 weeks after the last DMH injection (228 ± 39 *vs* 380 ± 55, $P < 0.05$, $n = 4$ rats per group). This significant increase in ACF between 1 and 5 weeks after the last DMH injection indicates that the total number of ACF continued to increase in the absence of additional carcinogen adminstration.

### *Distribution of different sized ACF along the length of the colon*

The average numbers of ACF of different sizes found in each centimetre along the length of the colon (expressed per 1.25 $cm^2$ of pinned-out colonic luminal surface area) were scored in rats (four per group) killed either 1 week or 5 weeks after the last of the eight weekly injections of DMH. The results are illustrated in Figure 2a and b. Most of the ACF of all size classes occurred in the distal half of the colon with a unimodal peak of the ACF occurring between the sites of the two most distal ALN. Few ACF occurred in the proximal half of the colon and no ACF occurred in the most proximal quarter of the colon.

### *Correlation analyses between (1) the distribution of ACF at 1 or at 5 weeks after the last DMH injection: (2) the distribution of tumours at 24 weeks after the last DMH injection; and (3) the distribution of pre-existing ALN along the length of the colon*

Figure 2c illustrates the distribution of adenomas and the distribution of AC along the length of the colon scored 24 weeks after the last of the eight weekly injections of DMH ($n = 335$ tumours in 402 rats.) Histological examination identified only 4.8% of all colon tumours as adenomas. There were three peaks in the distribution of AC along the length of the colon. The numerical distribution of adenomas along the length of the colon was not the same as the distribution of AC; specfically, 81% (13/16) of the adenomas were located between the sites of the distal two ALN but most of the ACs were located within the confines of the three sites of ALNs.

Table I presents the data on the distribution of 2640 ACF (from eight rats) and 335 tumours (from 402 rats) along the length of the colon of the DMH-treated rats. Linear regression analyses were performed on the data in Table I to determine if the numerical distribution of ACF (scored at 1 week or 5 weeks after the last DMH injection) correlated significantly with the numerical distribution of either adenomas or of ACs along the length of the colon (scored at 24 weeks after the last injection of DMH). The results of linear regression analyses indicated that the number of ACF along the length of the colon (counted at either 1 week or 5 weeks after the last DMH injection) did not correlate significantly ($P < 0.05$) with the number of adenomas or with the number of AC found along the length of the colon at 24 weeks after the last DMH injection.

To assess further the relationships between the distributions of ACF, ACs (of two morphological types, either polypoid or sessile) and ALNs along the length of the colon of DMH-treated Sprague–Dawley rats, the ACF data

**Figure 2** Distribution of ACF (classified as to the number of crypts involved, i.e. 1, 2, 3, etc. as indicated) of adenomas, of ACs and ALNs along the length of the colon of rats killed (**a**) 1, (**b**) 5 or (**c**) 24 weeks after the last eight weekly injections of DMH. The numbers of different-sized ACF were scored in two groups with four rats in each group; a total of 2640 ACF were counted. The per cent of the total number of adenomas and of AC in DMH-treated rats is plotted as a function of distance from the anus to ileum (data from Table I). All grossly visible tumours were classified by histology as an adenoma or as an AC. The tumour data were compiled from 335 tumours found in 402 Sprague–Dawley rats. The location of ALNs in the rat colon (determined from 20 non-DMH-treated rats) was measured as the distance from the anus to the distal end and to the proximal end of each ALN. The solid portion of the bar spans the distance between the mean of the distal end and the mean of the proximal end of each ALN, the open portion of the bar indicates one standard deviation (s.d.) from that mean. The mean ± 1 s.d. of the distal and proximal ends of each ALN (in per cent of total distance from the anus to the ileum) were as follows: distal ALN, 16.5 ± 1.29 to 21.9 ± 2.2; middle ALN, 51.7 ± 5.7 to 56.0 ± 5.9; and proximal ALN 86.5 ± 5.7 to 90.1 ± 6.3. No solitary lymphoid nodules were seen outside the locations indicated by the bars for ALN. This illustrates congruence in the occurrence of DMH-induced ACs and the locations of ALN in the rat, but lack of congruence in the distribution of ACF and the distribution of ACs.

collected from the experiments reported herein (collected at 1 week and at 5 weeks after eight subcutaneous injections of DMH) were correlated with published data which provided the distribution of tumours and ALNs (Nauss *et al.*, 1984) collected 4–14 months after a series of intragastric doses of DMH. The results of linear regression analyses between these data sets (as listed in the different columns of Table II) indicate: (1) that the distribution of ALN found at the time of sacrifice of the DMH-treated rats in the previous study (Nauss *et al.*, 1984) was significantly correlated ($P<0.05$) with the distribution of the more numerous sessile ACs but was not significantly correlated ($P>0.05$) with the distribution of the less numerous polypoid ACs; and (2) that the number of ACF (found at either 1 week or 5 weeks after the last exposure to DMH) did not correlate ($P>0.05$) with the distributions of either sessile or polypoid ACs along the length of the colon. The finding from the regression analyses between the two sets of experimental data must be tempered by the fact that the data of Nauss *et al.* (1984) were obtained on rats given DMH via intragastric gavage whereas the ACF data are from rats given DMH via subcutaneous injections. This and other differences between experiments makes the analyses less stringent than the analyses of data presented in Table I (data collected using the same protocol).

### Discussion

A main finding of this study is that the distribution of ACF along the length of the colon of carcinogen (DMH)-treated rats is not the same as the distribution of carcinogen-induced ACs which form in rats; thus, neither the number nor the size of ACF could be expected to directly predict the occurrence of AC. The lack of correlation between ACF and AC and the significant correlation between ALN and AC indicates that it is the enhancement of carcinogenesis due to the presence of ALN, not the numbers or distribution pattern of ACF, which is the main determinant of the distribution of ACs in the carcinogen-treated rats. The main reasons why the numbers and/or sizes of ACF have not been shown to predict the incidence of colonic AC reliably may be that the promotional effect of the ALNs is quite specific to the formation of endophytic ACs and/or that the promotional effect of the ALNs is localised to a relatively small proportion of the total luminal surface area of the colon of rats where ALNs occur. In this regard, it has been reported that about 70% of rat colonic ACs are endophytic or sessile ACs (Nauss *et al.*, 1984; Martin *et al.*, 1986; Hardman and Cameron, 1994) that are found in association with the small areas of ALNs.

It is worth noting that visible ALNs were observed in the

**Table I** Distribution of aberrant crypt foci (ACF), adenomas and adenocarcinomas along the length of the colon of Sprague–Dawley rats sacrificed at 1 week, at 5 weeks or at 24 weeks after the last eight weekly injections of dimethylhydrazine[a]

| Percentage distance from anus to ileum | Average total number of ACF 1.25 $cm^{-2}$ per rat n=1120, 4 rats (1 week) | n=1520, 4 rats (5 weeks) | % of total tumours: Adenomas n=16, 402 rats (24 weeks) | Adenocarcinomas n=319, 402 rats (24 weeks) |
|---|---|---|---|---|
| 15 | 22.0 | 42.5 | 0.6 | 22.9 |
| 20 | 29.5 | 40.8 | – | – |
| 25 | 37.5 | 57.0 | 0.0 | 25.4 |
| 30 | 42.0 | 77.5 | – | – |
| 35 | 49.0 | 58.3 | 1.5 | 4.5 |
| 40 | 38.0 | 48.5 | – | – |
| 45 | 29.0 | 31.8 | 2.1 | 16.7 |
| 50 | 20.5 | 15.0 | – | – |
| 55 | 8.0 | 6.3 | 0.3 | 7.5 |
| 60 | 4.5 | 2.8 | – | – |
| 65 | 0.5 | 1.0 | 0.0 | 1.5 |
| 70 | 0.0 | 0.8 | – | – |
| 75 | 0.0 | 0.0 | 0.0 | 3.3 |
| 80 | 0.0 | 0.0 | – | – |
| 85 | 0.0 | 0.0 | 0.3 | 10.2 |
| 90 | 0.0 | 0.0 | – | – |
| 95 | 0.0 | 0.0 | 0.0 | 3.3 |

[a]The results of linear regression analyses between the values listed in the different columns are presented in the text.

**Table II** Distribution of aberrant crypt foci (data from the study presented in this report) of adenocarcinomas (divided into polypoid and sessile types) and of aggregates of lymphoid nodules (the adenocarcinoma and ALN data are from experiment no.3 in the report of Nauss *et al.* 1984) along the length of the colon of Sprague–Dawley rats (sacrificed at times given in parenthesis) after the last of a series of five or eight weekly exposures (either intragastric or subcutaneous injections) to dimethylhydrazine[a]

| Percentage distance from anus to ileum[b] | Aberrant crypt foci number 1.25 $cm^{-2}$ n=4 rats (1 week) | n=4 rats (5 weeks) | Adenocarcinomas: Polypoid n=159 rats (4–14 months) | Sessile n=159 rats (4–14 months) | Aggregates of lymphoid nodules n=159 rats (4–14 months) |
|---|---|---|---|---|---|
| 6.3 | 4.4 | 8.5 | 2 | 1 | 2 |
| 18.8 | 31.0 | 47.2 | 4 | 11 | 80 |
| 31.3 | 42.2 | 64.0 | 4 | 2 | 18 |
| 43.8 | 27.4 | 28.5 | 13 | 8 | 44 |
| 56.3 | 5.2 | 3.8 | 13 | 24 | 51 |
| 68.8 | 0 | 0.3 | 2 | 0 | 15 |
| 81.3 | 0 | 0 | 6 | 40 | 67 |
| 93.8 | 0 | 0 | 3 | 14 | 26 |
| 100.0 | 0 | 0 | 1 | 4 | 0 |

[a]The results of linear regression analyses between the values listed in the different columns are presented in the text. [b]Data of Nauss *et al.* (1984) were collected from 3 cm length segments. The mean (in per cent of the total distance from the anus to the iluem) of each segment is reported in this table.

proximal quarter of the colon in the majority of the rats, but that none of the 2640 ACF were found in this proximal quarter of the colon at either 1 or 5 weeks after the last DMH injection. As the distribution of ACF was not scored in the proximal portion of the colon at 24 weeks after the last DMH injection, it cannot be claimed with certainty that no ACF ever arise in this region of the colon. However, if an ACF arises in this segment of the colon it must be considered an extremely rare event. Thus, it seems highly unlikely that ACs which do form in this proximal quarter of the colon could have arisen via an ACF precursor pathway. The likely explanation for AC genesis in this proximal segment of the colon is via the *de novo* pathway of AC formation.

If one assumes, for the sake of discussion, that ACF are the sole precursor lesions to ACs, then one can calculate the expected conversion of ACF to ACs. For example, based on the data in Table I, the average number of ACF per rat at 5 weeks was 380; this compares with an average of 0.79 ACs per rat at 24 weeks after the last DMH injection. Thus, only a small percentage (0.2%) of the ACF present at 5 weeks could have evolved into ACs present at 24 weeks. Similar calculations done at about 35% of the distance from the anus to the ileum results in an even lower percentage of possible conversions of ACF to ACs (0.06%) in the area between the two distal ALNs. This latter estimate of possible conversion of putative premalignant foci to malignant foci in an area of the colon without putative promotion effects from the lymphatic nodules compares with an estimate of 0.02% for conversion of carcinogen-induced premalignant aberrant liver foci to malignant tumours in the rat liver (Williams and Watanbe, 1978). Based on these calculations, it may be concluded that progression from a carcinogen-altered foci to a malignant tumour is rare both in the liver and in the colon of rats. However, the calculated conversion of ACF to ACs (based on the data in Table I) is 4- to 5-fold higher in the sites of the two distal ALNs in the rat colon than in the area between the two distal ALN sites. Thus, ALNs are either promotional to the rare conversion of ACF to become ACs and/or are promotional to the *de novo* formation of ACs. These two possibilities are not mutually exclusive in the distal colon, but either possibility indicates a promotional role for ALNs in colon carcinogenesis. Calculation of the potential ACF to AC conversion percentages in the proximal quarter of the colon was not possible as no ACF could be found in the proximal quarter of the colon.

The above findings are not intended to convey to the reader that measurement of the numbers, size and distribution of ACF in rats is of little or no value as a biomarker of colon carcinogenesis. The number of ACF found in the colon is undoubtedly an indicator of exposure to the known colon carcinogens so far tested (McLellan and Bird, 1988; Tudek *et al.*, 1989), and in this regard ACF can be said to reflect risk of colon cancer. But because neither the number nor the size of ACF has proved a significant predictor of AC formation along the length of the colon in the rat model system (this report) or in the mouse (Carter *et al.*, 1994), the use of the total numbers or size of ACF in the colon of rats is not necessarily a valid indicator of the risk of malignant colon cancer. Indeed, there are now several reports from independent studies which fail to confirm numbers of ACF as a reliable indicator of cancer risk throughout the colon of rats (Hardman *et al.*, 1991; Magnuson and Bird, 1993; Magnuson *et al.*, 1993; Caderni *et al.*, 1995; Thorup *et al.*, 1994).

In conclusion, the results from the present and from past reports (Nauss *et al.*, 1984; Shimamoto and Vollmer, 1987; Martin *et al.*, 1986; Hardman and Cameron, 1994; Carter *et al.*, 1994; Shamsuddin and Hogan, 1984) indicate a strong promotional role for lymphoid nodules in colon carcinogenesis. That ACs can arise along a pathway that does not pass through an ACF stage seems highly likely in the proximal quarter of the colon of the rat (data in this report). It also seems that there is no direct proof that any aberrant crypt foci progress to become malignant cancer, nor is there direct proof to the contrary. The question remains: do ACF represent true premalignant lesions which on rare occasion progress via a multistep process to become ACs or do ACF and colon AC represent end points of two parallel but independent pathways resulting as a consequence of a common colon cancer initiation (Thorup *et al.*, 1994; Jen *et al.*, 1994; Smith *et al.*, 1994; Yamashita *et al.*, 1995; Pretlow, 1995)? Serial observations using endoscopic procedures on humans and on animal models followed by terminal histology may eventually provide direct evidence on the fate of ACF. Regardless of the outcome, the fact remains that the numbers and the multiplicity (size) of ACF are currently being used as biomarkers of colon cancer risk (Lam and Zhang, 1991; Periera and Khoury, 1991; O'Riordan *et al.*, 1991; Wargovich *et al.*, 1992; Rao *et al.*, 1993; Zhang *et al.*, 1992; Deschner *et al.*, 1990; Pereira *et al.*, 1994) but interpretation of the results of intervention studies that use ACF as biomarkers of efficacy of intervention must take into account the questionable value of ACF numbers and sizes as valid predictors of colon cancer risk.

**Acknowledgements**
This research was supported in part by the American Cancer Society BC-641, the American Institute for Cancer Research, MBRS/NIH 506 MG 8170 and VA-93-0001.

### References

AMERICAN INSTITUTE OF NUTRITION. (1977). Report of the American Institute of Nutrition *ad hoc* committee on standards for nutritional studies. *J. Nutr.*, **107**, 1340–1348.

BLAND PW AND BRITTON DC. (1984). Morphological study of antigen-sampling structures in the rat large intestine. *Infect. Immun.*, **43**, 693–699.

CADERNI G, GIANNINI A, LANCIONI L, LUCERI C, BIGGERI A AND DOLARA P. (1995). Characterisation of aberrant crypt foci in carcinogen-treated rats: association with intestinal carcinogenesis. *Br. J. Cancer*, **71**, 763–769.

CARTER JW, LANCASTER HK, HARDMAN WE AND CAMERON IL. (1994). Distribution of intestine-associated lymphoid-tissue, aberrant crypt foci and tumours in the large bowel of 1,2-dimethylhydrazine-treated mice. *Cancer Res.*, **54**, 4304–4307.

DESCHNER EE, LYTLE JS, WONG G, RUPERTO JF AND NEWMARK HL. (1990). The effect of dietary omega-3 fatty acids (fish oil) on azoxymethanol-induced focal areas of dysplasia and colon tumour incidence. *Cancer*, **66**, 2350–2356.

HARDMAN WE AND CAMERON IL. (1994). Colonic crypts located over lymphoid nodules of 1,2-dimethylhydrazine-treated rats are hyperplastic and at high risk of forming adenocarcinomas. *Carcinogenesis*, **15**, 2353–2361.

HARDMAN WE, CAMERON IL, HEITMAN DW AND CONTRERAS E. (1991). Demonstation of the need for end point validation of putative biomarkers: failure of aberrant crypt foci to predict colon cancer incidence. *Cancer Res.*, **51**, 6388–6392.

JEN J, POWELL SM, PAPADOPOULOS N, SMITH KJ, HAMILTON SR, VOGELSTEIN B AND KINZLER KW. (1994). Molecular determinants of dysplasia in colorectal lesions. *Cancer Res.*, **54**, 5523–5526.

LAM LKT AND ZHANG J. (1991). Reduction of aberrant crypt formation in the colon of CF1 mice by potential chemopreventive agents. *Carcinogenesis*, **12**, 2311–2315.

MCLELLAN EA AND BIRD RP. (1988). Aberrant crypts: potential preneoplastic lesions in the murine colon. *Cancer Res.*, **48**, 6187–6192.

MAGNUSON BA AND BIRD RP. (1993). Reduction of aberrant crypt foci induced in rat colon with azoxymethane or methylnitrosourea by feeding cholic acid. *Cancer Lett.*, **68**, 15–23.

MAGNUSON BA, CARR I AND BIRD RP. (1993). Ability of aberrant crypt foci characteristics to predict colonic tumour incidence in rats fed cholic acid. *Cancer Res.*, **53**, 4499–4504.

MARTIN MS, HAMMAN A AND MARTIN F. (1986). Gut-associated lymphoid tissues and 1,2-dimethylhydrazine intestinal tumours in the rat: an histological and immunoenzymatic study. *Int. J. Cancer*, **38**, 75–80.

NAUSS KM, LOCNISKAR M AND NEWBERNE PM. (1984). Morphology and distribution of 1,2-dimethylhydrazine dihydrochloride-induced colon tumours and their relationship to gut-associated lymphoid tissue in rats. *J. Natl. Cancer Inst.*, **73**, 915–921.

NIE N, HULL CH, JENKINS JG, STEINBRENNER K AND BRENDT DH. (1983). *SPSS$^{x}$ Users' Guide*, McGraw-Hill: New York.

O'RIORDAN MA, PRETLOW TG, STELLATO TA AN PRETLOW TP. (1991). Effect of selenium on the induction of aberrant crypts in the colons of rats treated with azoxymethane. *Proc. Am. Assoc. Cancer Res.*, **32**, 147.

PERIERA MA AND KHOURY MD. (1991). Prevention by chemopreventive agents of azoxymethane-induced foci of aberrant crypts in rat colon. *Cancer Lett.*, **61**, 27–33.

PEREIRA MA, BARNES LH, RASSMAN VL, KELLOFF GV AND STEELE VE. (1994). Use of azoxymethane-induced foci of aberrant crypts in rat colon to identify potential cancer chemopreventive agents. *Carcinogenesis*, **15**, 1049–1054.

PRETLOW TP. (1995). Aberrant crypt foci and K-*ras* mutations: earliest recognised players or innocent bystanders in colon carcinogenesis. *Gastroenterology*, **108**, 600–609.

RAO CV, DESAI D, SIMI B, KULKARNI N, AMIN S AND REDDY BS. (1993). Inhibitory effect of caffeic acid esters on modulation of azoxymethane-induced early events and aberrant crypt foci formation in rat colon. *Proc. Am. Assoc. Cancer Res.*, **34**, 165(Abstract).

SHAMSUDDIN AM AND HOGAN ML. (1984). Large intestinal carcinogenesis. II. Histogenesis and unusual features of low-dose azoxymethane-induced carcinomas in F344 rats. *J. Natl. Cancer Inst.*, **73**, 1297–1305.

SHIMAMOTO F AND VOLLMER E. (1987). Changes in intestinal mucosa above lymph follicles during carcinogenesis in rats. *J. Cancer Res. Clin. Oncol.*, **113**, 41–50.

SMITH AJ, STERN HS, PENNER M, HAY K, MITRI A, BAPAT BV AND BALLINGER S. (1994). Somatic *APC* and K-*ras* codon 12 mutations in aberrant crypt foci from human colons. *Cancer Res.*, **54**, 5527–5530.

THORUP I, MEYER O AND KRISTIANSEN E. (1994). Influence of a dietary fiber on development of dimethylhydrazine-induced aberrant crypt foci and colon tumour incidence in wistar rats. *Nutr. Cancer*, **21**, 177–192.

TUDEK B, BIRD RP AND BRUCE WR. (1989). Foci of aberrant crypts in the colons of mice and rats exposed to carcinogens associated with foods. *Cancer Res.*, **49**, 1236–1240.

WARGOVICH MJ, HARRIS C, CHEN C, PALMER C, STEELE VE AND KELLOFF GJ. (1992). Growth kinetics and chemoprevention of aberrant crypts in the rat colon. *J. Cell. Biochem.*, **16G**, 51–54.

WILLIAMS GM AND WATANBE K. (1978). Quantitative kinetics of development of n-2-fluorenylacetamide induced, altered (hyperplastic) hepatocellular foci resistant to iron accumulation and of their reversion or persistence following removal of carcinogen. *J. Natl. Cancer. Inst.*, **61**, 113–121.

YAMASHITA N, MINAMOTO T, OCHIAI A, ONDA M AND ESUMI H. (1995). Frequent and characteristic K-*ras* activation and absence of p53 protein accumulation in aberrant crypt foci of the colon. *Gastroenterology*, **108**, 434–440.

ZHANG XM, STAMP D, MINKIN S, MEDLINE A, CORPET DE, BRUCE WR AND ARCHER MC. (1992). Promotion of aberrant crypt foci and cancer in rat colon by thermolysed protein. *J. Natl. Cancer Inst.*, **84**, 1026–1030.